# Building the social prescribing student movement in Canada

**DOI:** 10.24095/hpcdp.44.6.06

**Published:** 2024-06

**Authors:** Caitlin Muhl, Le-Tien Bhaskar, Michelle Ruhigisha, Ellen McGarity-Shipley

**Affiliations:** 1 Canadian Social Prescribing Student Collective, Toronto, Ontario, Canada; 2 Faculty of Health Sciences, Queen’s University, Kingston, Ontario, Canada; 3 Department of Health Research Methods, Evidence, and Impact, McMaster University, Hamilton, Ontario, Canada; 4 Faculty of Applied Health Sciences, Brock University, St. Catharines, Ontario, Canada

**Keywords:** social movement, social prescribing, students

**Highlights**

- A global network of student champions has emerged to build the social prescribing student movement, with student groups in seven countries, including Canada.
- The Canadian Social Prescribing Student Collective was established in 2022.
- Much progress has been made in building the social prescribing student movement in Canada, but there is a lot of work to be done, which calls for action by students, staff in health care and community organizations, and faculty and administration at postsecondary institutions.
- Collective efforts to build the social prescribing student movement in this country will not only shape the wider social prescribing movement, but also the future of our health system.

## Introduction

Social prescribing is defined as “a means for trusted individuals in clinical and community settings to identify that a person has nonmedical, health-related social needs and to subsequently connect them to nonclinical supports and services within the community by co-producing a social prescription—a nonmedical prescription, to improve health and well-being and to strengthen community connections.”^1^^,p.9^ Globally, there is growing interest in social prescribing as a holistic approach to health and well-being, with almost 30countries involved in the social prescribing movement.^2^ In Canada, great strides are being made in social prescribing research, policy and practice, with all of this work being supported by the Canadian Institute for Social Prescribing.^3^

Alongside the rapid expansion of the social prescribing movement around the world, a global network of student champions has emerged to build the social prescribing student movement. In the United Kingdom (UK), where the social prescribing movement began, students have been heavily involved right from the start. In 2017, the UK National Social Prescribing Student Champion Scheme was established by Dr. Bogdan Chiva Giurca—a medical student at the time—to enable students to get involved in learning about, teaching and promoting social prescribing.^4^ Since then, more than 350 student champions have engaged with over 20000 learners across the UK.^5^

Over the past few years, the social prescribing student movement has expanded to several other countries, including Australia, Japan, Portugal, Singapore and the United States (US).^6^ In 2021, these student groups came together to develop the Social Prescribing International Student Movement Framework, which served as a call to action and a guidebook for student champions across the globe.^6^ This framework caught the attention of students in Canada who shared a passion for social prescribing and a desire to bring the social prescribing student movement to this country, which led to the establishment of the Canadian Social Prescribing Student Collective.

In this commentary, we outline the importance of building the social prescribing student movement in Canada, give an overview of the Canadian Social Prescribing Student Collective, provide examples of ways in which students are contributing to social prescribing efforts across the nation, and put out a call to action to advance the social prescribing student movement in this country.

## The importance of building the social prescribing student movement in Canada

Throughout history, students have been a driving force behind social movements.^7^ A recent example of this is the critical role that student activists have played in shaping the Black Lives Matter movement.^8^ It follows that the social prescribing movement stands to benefit from the power of students to foster social change. Looking at the Canadian context, anecdotal evidence suggests that students are eager to support the movement, and members of the social prescribing community agree that student involvement in the movement is fundamental to its success. 

But this is not only about the impact of students on the movement—this is also about the impact of the movement on students. This is about empowering today’s learners, who will become tomorrow’s leaders. With evidence to suggest that up to 50% of primary care visits are for nonmedical issues,^9^ we must move beyond the biomedical model by shifting care upstream to address the nonmedical factors that determine 80% to 90% of health and well-being.^10^-^12^ The case for this shift has never been clearer given the perfect storm of a pandemic,^13^ an aging global population^14^ and an estimated global shortage of 18 million health workers (20% of the global workforce) by 2030.^15^ In the wise words of Hamaad Khan, a medical student and social prescribing champion in the UK, “We must ask ourselves, where is the health in our health system, and where is the care in our health care?”^16^ We are at a crisis point, but there is hope for the future; by instilling the values of social prescribing in our students, we will empower them to create health in our health system and deliver care in our health care. This is what we hope to achieve by building the social prescribing student movement in Canada.

## The Canadian Social Prescribing Student Collective

The Canadian Social Prescribing Student Collective was launched in March 2022. Our mission is to build the social prescribing student movement across Canada. We are guided by our four values: (1) collaborate, (2) educate, (3) advocate and (4)innovate. We have over 350 members, who represent more than 35 postsecondary institutions in British Columbia, Alberta, Saskatchewan, Ontario, Quebec, New Brunswick, Nova Scotia, Prince Edward Island and Newfoundland and Labrador. Our members include undergraduate students, graduate students and college students, with representation from a variety of different programs (i.e. health sciences, kinesiology, medicine, nursing, pharmacy, psychology, public health, social work, etc.). 

In addition to our members, we have over 20 academic and community partners. As an online community, we communicate with our members and partners through email, newsletter, Slack, and Zoom meetings, and we engage with the wider social prescribing community through our webpage, our social media accounts, webinars, presentations and conference sessions. We are affiliated with the Canadian Institute for Social Prescribing, which ensures alignment between the social prescribing student movement and the wider social prescribing movement in Canada.

Our efforts to build the social prescribing student movement span local, national and international levels. Locally, we have chapters at postsecondary institutions. Nationally, we convene through general meetings, as well as through five working groups with specific focus areas: (1)research, (2) policy, (3) practice, (4) knowledge translation and (5) medicine. Internationally, we represent Canada on the Global Social Prescribing Student Council, which brings together the leaders of social prescribing student groups around the world to advance the global social prescribing student movement.

We recently conducted a member experience survey, which revealed that students are benefiting from being involved in our group. When asked about their participation, 82.3% of our members agreed that this group has improved their knowledge of social prescribing, and 88.2% agreed that this group has helped them to connect with other students who are interested in social prescribing. Looking to the future, we hope to expand our efforts, grow our membership, develop a better understanding of the effectiveness of our efforts, and contribute to the social prescribing evidence base by examining relevant areas that have yet to be explored, such as what motivates students to become involved in the social prescribing student movement.

## Student contributions to social prescribing efforts in Canada

Students first became involved in social prescribing efforts in Canada several years before the launch of the Canadian Social Prescribing Student Collective. For example, Canada’s first social prescribing pilot (2018–2020) benefited from the support of practicum students.^17^ We note that social prescribing programs have been harnessing the potential of students to act as connectors for more than a decade.^18^,^19^ In British Columbia, Basics for Health Society was established in 2012 as a way for health care and community organizations to address patients’ social needs through the use of trained student volunteers, who connect patients to community resources.^18^ In Ontario, the NORTH (Navigating Ottawa Resources To Improve Health) Clinic, run by medical and law students at the University of Ottawa, was founded in 2018.^19^ Patients with social needs are referred from health caresettings to the NORTH Clinic, where trained student volunteers connect the patients to community resources. These programs not only serve to address patients’ social needs and improve health equity for underserved communities, but also to enrich the educational experiences of students through experiential learning opportunities.

Since the launch of the Canadian Social Prescribing Student Collective, several student-led initiatives have emerged as a direct result of the student community that has been cultivated. Locally, our Brock University Chapter is collaborating with student health services to implement social prescribing on campus. Elsewhere in Ontario, our University of Toronto Chapter hosted the first social prescribing student conference in the country. Nationally, our research working group is conducting a scoping review on social prescribing and students,^20^ and our policy working group recently developed a policy brief to advocate for the importance of social prescribing in supporting student mental health on campus.

Apart from the work that is happening through the Canadian Social Prescribing Student Collective, students are contributing to social prescribing research, policy and practice through various academic pursuits, including thesis work, practicums, traineeships, internships, research assistantships and co-op placements. 

For example, practicum students in Ontario at the University of Guelph are working with the social prescribing program at Guelph Community Health Centre; trainees in Quebec at McGill University are supporting efforts to implement social prescribing in primary care clinics; and students across the country are supporting the work that is happening at the Canadian Institute for Social Prescribing through internships and research assistantships. 

Additionally, students are supporting the social prescribing movement through paid and unpaid roles that are distinct from, but complementary to, their academic activities. For example, students are coordinating social prescribing programs at organizations such as Fraser Health in British Columbia and the Vanier Social Pediatric Hub in Ontario, and students across the country are volunteering for programs such as the Canadian Red Cross Friendly Calls Program and the Student–Senior Isolation Prevention Partnership to fulfill social prescriptions for people who are feeling socially isolated and lonely. 

All of these experiences allow students to apply what they have learned in the classroom about health promotion, upstream thinking and the power of community.

## A call to action

While it is important to celebrate the progress that has been made in building the social prescribing student movement thus far, there is a lot of work to be done. This is a call to action for students, staff in health care and community organizations, and faculty and administration at postsecondary institutions to support the advancement of the social prescribing student movement in Canada.

**1. We call on students to join the Canadian Social Prescribing Student Collective and to support social prescribing research, policy and practice through academic (i.e. thesis work, practicums, traineeships, internships, research assistantships, co-op placements, etc.) and nonacademic avenues (i.e. paid and unpaid roles).**

You can sign up here: www.socialprescribing.ca/student-collective.

**2. We call on staff in health care and community organizations and faculty and administration at postsecondary institutions to connect with us and to increase the level and type of student involvement in social prescribing efforts (e.g. connector role, program development, program evaluation, research) through academic and nonacademic avenues.**

Whether you simply wish to receive our newsletter, or want to explore opportunities for student engagement, you can sign up here: www.socialprescribing.ca/student-collective.

**3. We call on faculty and administration at postsecondary institutions to complement our efforts to educate students about social prescribing by integrating this concept into the curriculum for health professional programs (i.e. medicine, nursing, occupational therapy, pharmacy, physiotherapy, social work, etc.) through a combination of didactic teaching and experiential learning opportunities.**

Experiences in other countries reveal that student-led efforts to educate peers about social prescribing need to be supplemented with formal education through a combination of didactic teaching and experiential learning opportunities.^4^,^21^-^25^

Together, these actions will help to build the social prescribing student movement, which will not only shape the wider social prescribing movement, but also the future of our health system.

## Acknowledgements

We acknowledge Sonia Hsiung for her mentorship and for providing us with valuable feedback on this commentary. We thank Niloufar Aran for the contributions she made to the Canadian Social Prescribing Student Collective as a Co-Founder and Co-Lead, prior to her departure to study abroad.

## Conflicts of interest

The authors have no conflicts of interest to declare.

## Authors’ contributions and statement

CM—conceptualization.

CM—writing—original draft.

CM, LB, MR, EMS—writing—review and editing.

The content and views expressed in this article are those of the authors and do not necessarily reflect those of the Government of Canada.
